# Association Between Genetically Predicted 25‐Hydroxyvitamin D and Type 2 Diabetes Risk in South Asians: An Exploratory Two‐Sample Mendelian Randomization Study

**DOI:** 10.1002/edm2.70359

**Published:** 2026-10-22

**Authors:** Adina Zaka, Suleman Khan, Asim Shah, Aizaz Anwar Khalid, Ibrahim Khalil, A. B. M. Kamrul‐Hasan

**Affiliations:** ^1^ Rahbar Medical and Dental College Lahore Pakistan; ^2^ Khyber Medical College Peshawar Pakistan; ^3^ Peshawar Medical College Peshawar Pakistan; ^4^ Department of Internal Medicine Dhaka Medical College and Hospital Dhaka Bangladesh; ^5^ Department of Endocrinology Mymensingh Medical College Mymensingh Bangladesh

**Keywords:** causal inference, GWAS, mendelian randomization, pleiotropy, south Asian, Type 2 diabetes, vitamin D

## Abstract

**Background:**

Vitamin D deficiency and Type 2 diabetes (T2D) are highly prevalent in South Asian populations. Observational studies report inverse associations between serum 25‐hydroxyvitamin D [25(OH)D] and T2D risk, but these findings may reflect confounding or reverse causation. We used Mendelian randomization (MR) to assess the association between genetically predicted 25(OH)D and T2D risk in South Asians.

**Methods:**

We conducted a two‐sample MR analysis using publicly available OpenGWAS summary statistics. One variant (rs3755967) met the conventional genome‐wide significance threshold (*p* < 5 × 10^−8^), while two additional variants (rs2459139 and rs72744878) met an exploratory threshold (*p* < 1 × 10^−6^). The three variants served as instruments for serum 25(OH)D in a South Asian GWAS (ukb‐e‐30890_CSA; *n* = 7380; mean *F*‐statistic = 29.6; total *R* ^2^ = 1.06%). The primary outcome was BMI‐unadjusted T2D (ebi‐a‐GCST90093109; *n* = 50,533; 16,677 cases), with a BMI‐adjusted GWAS used for sensitivity analysis. The primary estimator was inverse‐variance weighted (IVW), supported by weighted‐median, MR‐Egger, simple‐mode, and weighted‐mode analyses. Heterogeneity and directional pleiotropy were assessed using Cochran's *Q* and the MR‐Egger intercept, respectively. Partial participant overlap could not be excluded because UK Biobank contributed to both datasets.

**Results:**

The primary IVW analysis showed no statistically significant association between genetically predicted 25(OH)D and T2D risk (*β* = 0.058, SE = 0.120, *p* = 0.630; OR = 1.06, 95% CI: 0.83–1.36). Weighted‐median and mode‐based estimates were also non‐significant. The MR‐Egger estimate was highly imprecise (OR = 32.0, 95% CI: 0.11–9103) because only three instruments were available. No significant heterogeneity or directional pleiotropy was detected, although these tests had limited power. The BMI‐adjusted sensitivity analysis produced similar findings.

**Conclusions:**

We found no statistically significant evidence of a linear association between genetically predicted serum 25(OH)D and T2D risk. However, the small exposure GWAS, limited instrument set, exploratory instrument threshold, and possible sample overlap mean that small‐to‐moderate causal effects cannot be excluded. Larger South Asian GWAS are needed for more definitive inference.

## Graphical abstract

Using two‐sample Mendelian randomization, we found no statistically significant association between genetically predicted 25‐hydroxyvitamin D and Type 2 diabetes risk in South Asians. However, the limited exposure GWAS, three‐instrument set, exploratory instrument threshold, and possible sample overlap warrant cautious interpretation and larger South Asian GWAS.

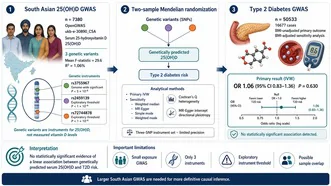

## 1 Introduction

Vitamin D deficiency is one of the most widespread nutritional disorders globally, with South Asian populations being disproportionately affected. A systematic review and meta‐analysis pooling South Asian data estimates the prevalence of Vitamin D deficiency in Pakistan at approximately 73% (95% CI: 63%–83%), although with substantial between‐study heterogeneity (*I* ^2^ ≈ 99%); estimates in individual cohorts vary widely, largely reflecting dietary insufficiency, cultural practices that reduce sun exposure, and lower cutaneous Vitamin D synthesis associated with darker skin pigmentation [1, 2]. At the same time, Pakistan faces one of the highest burdens of Type 2 diabetes (T2D) worldwide, affecting approximately 26% of adults, or more than 33 million people. This overlap makes the Pakistani population particularly important for research focused on metabolic disease prevention and risk modification [3].

There are biologically plausible mechanisms through which Vitamin D may influence the development of T2D. Vitamin D receptors (VDR) are expressed in pancreatic beta cells, suggesting a role in beta‐cell function and insulin secretion [4, 5]. Experimental evidence also indicates that Vitamin D may improve peripheral insulin sensitivity and reduce systemic inflammation, both of which are central to T2D pathogenesis [4, 5]. Consistent with these mechanisms, observational studies across diverse populations have reported inverse associations between serum 25‐hydroxyvitamin D [25(OH)D] concentrations and T2D risk. However, these associations may be affected by residual confounding from lifestyle factors, including obesity, physical inactivity, and poor diet. Reverse causation may also contribute, as individuals with T2D or diabetes‐related complications may spend less time outdoors, resulting in lower Vitamin D levels. Therefore, biological plausibility and observational evidence alone cannot establish a causal relationship. Figure 1 shows the proposed biological mechanisms linking Vitamin D status with Type 2 diabetes pathophysiology.

**FIGURE 1:**
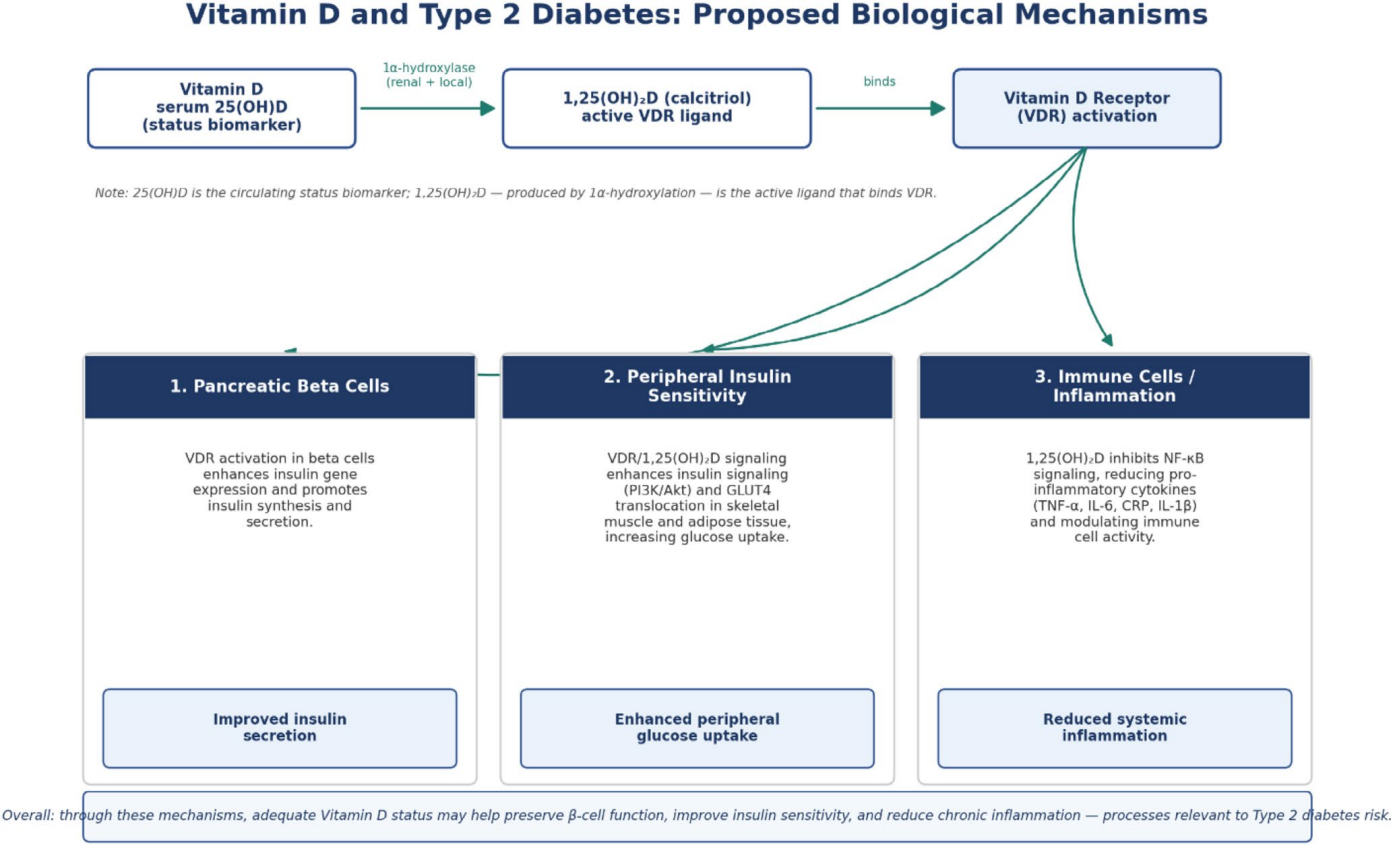
Proposed biological mechanisms linking Vitamin D status with Type 2 diabetes pathophysiology. Vitamin D, measured as serum 25‐hydroxyvitamin D [25(OH)D], is hydroxylated to the active hormone 1,25‐dihydroxyvitamin D [1,25(OH)2D, calcitriol], which is the principal ligand for the Vitamin D receptor (VDR); 25(OH)D itself is the circulating status biomarker rather than the direct VDR ligand. Through 1,25(OH)2D‐mediated VDR signalling and other pathways, Vitamin D status may influence glucose metabolism via three major biological mechanisms: activation of VDR in pancreatic beta cells, leading to improved insulin synthesis and secretion; enhancement of peripheral insulin sensitivity in skeletal muscle and adipose tissue through improved insulin signalling and glucose uptake; and reduction of systemic inflammation through modulation of immune‐cell activity and suppression of pro‐inflammatory cytokines. Together, these mechanisms suggest that Vitamin D may contribute to beta‐cell preservation, improved glucose homeostasis, and reduced inflammatory burden relevant to Type 2 diabetes development and progression. 25(OH)D, 25‐hydroxyvitamin D; CRP, C‐reactive protein; GLUT4, glucose transporter Type 4; IL‐1β, interleukin‐1 beta; IL‐6, interleukin‐6; NF‐κB, nuclear factor kappa B; T2D, Type 2 diabetes; TNF‐α, tumour necrosis factor‐alpha; VDR, Vitamin D receptor.

Mendelian randomization (MR) offers a useful approach to address these limitations by using germline genetic variants as instrumental variables [6, 7]. Since genetic variants are fixed at conception, MR analyses are less vulnerable to confounding and reverse causation than conventional observational studies. Several MR studies have investigated the relationship between Vitamin D and T2D, predominantly in populations of European ancestry, but their findings have been inconsistent [8]. Determining whether a causal relationship exists in South Asian populations is especially important for two reasons. First, both Vitamin D deficiency and T2D are highly prevalent in this region. Second, differences in genetic architecture, dietary patterns, adiposity profiles, and environmental exposures may limit the direct applicability of findings from European‐ancestry cohorts to South Asian populations.

With this rationale, we conducted a two‐sample MR study to evaluate the association between genetically predicted Vitamin D levels and T2D risk in South Asians. We used the largest available South Asian genome‐wide association study (GWAS) datasets for both Vitamin D and T2D. We also acknowledge at the outset that the available South Asian Vitamin D GWAS is substantially smaller than comparable European datasets, which may reduce instrument strength and limit the range of pleiotropy sensitivity analyses that can be performed. This limitation is addressed explicitly throughout the study.

## 2 Methods

### 2.1 Study Design

We employed a two‐sample Mendelian randomization design, in which single nucleotide polymorphism (SNP)‐exposure and SNP‐outcome associations were obtained from separate GWAS analyses. However, partial participant overlap could not be excluded: the exposure GWAS (ukb‐e‐30890_CSA) is drawn entirely from UK Biobank, and the outcome meta‐analysis (Loh et al. [9]) combined 16 South Asian cohorts, one of which was UK Biobank (imputed to the Haplotype Reference Consortium panel, distinct from the 1000 Genomes‐imputed cohorts). The exact number of overlapping UK Biobank participants could not be determined from the published report or its supplementary data, and is addressed as a limitation (Section 5). This approach is appropriate when a single large South Asian GWAS containing measurements of both Vitamin D and T2D is unavailable. The study is reported in accordance with the STROBE‐MR reporting guidelines [10]. Figure 2 shows the summarized study design.

**FIGURE 2:**
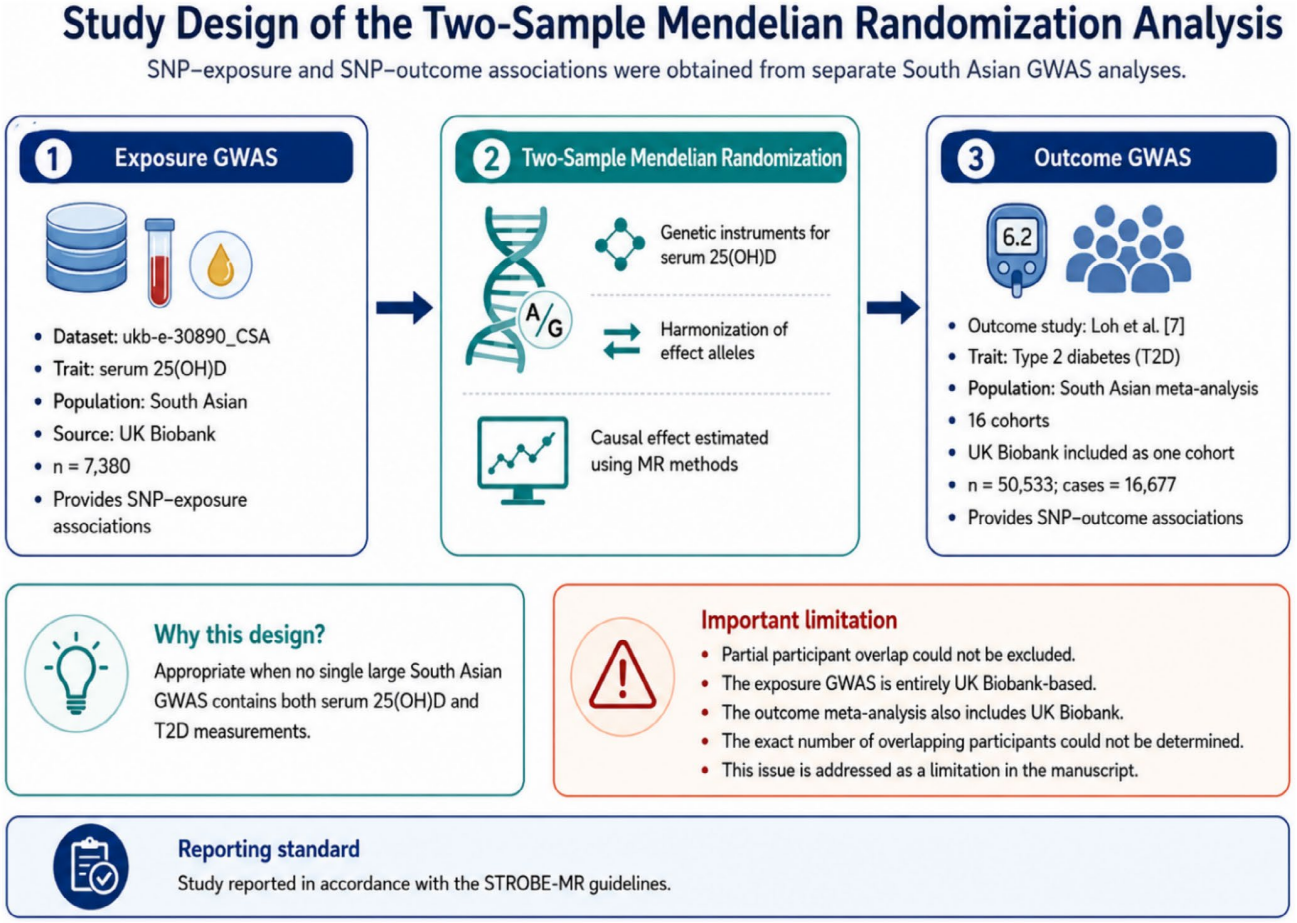
Study design of the two‐sample Mendelian randomization analysis. SNP–exposure associations for serum 25(OH)D were obtained from a South Asian UK Biobank GWAS, and SNP‐outcome associations for T2D were obtained from a separate South Asian meta‐analysis. The design also highlights the possibility of partial participant overlap and reporting in accordance with STROBE‐MR guidelines.

### 2.2 Data Sources

All GWAS summary statistics were obtained from the OpenGWAS database (gwas.mrcieu.ac.uk), a publicly curated repository of harmonized GWAS results as shown in Table 1. No individual‐level patient data were accessed. As this is a secondary analysis of publicly available summary‐level data, formal ethical approval was not required; all source GWAS were conducted under appropriate ethical protocols with informed consent from original participants.

**TABLE 1:** GWAS datasets used in this Mendelian Randomization study. Outcome T2D GWAS: Loh et al. [9].

Role	Dataset ID	Trait	Ancestry	Sample size
Exposure	ukb‐e‐30890_CSA	Vitamin D levels	South Asian	*n* = 7380
Outcome	ebi‐a‐GCST90093109	T2D (BMI‐unadjusted, primary)	South Asian	*n* = 50,533 (cases = 16,677)

Abbreviations: BMI, Body Mass Index; GWAS, Genome‐Wide Association Study; T2D, Type 2 Diabetes.

In the source GWAS, T2D cases were defined by a history of T2D or T2D treatment, fasting plasma glucose ≥ 7.0 mmol/L, 2‐h oral glucose tolerance test glucose ≥ 11.1 mmol/L, or HbA1c ≥ 6.5% [9]. The BMI‐unadjusted GWAS (ebi‐a‐GCST90093109) was used as the primary outcome, while the BMI‐adjusted GWAS (ebi‐a‐GCST90093110), based on the same T2D phenotype, was included as a pre‐specified sensitivity analysis. Because BMI adjustment could attenuate effects operating through adiposity, the adjusted analysis was interpreted as a sensitivity analysis rather than a replacement for the primary outcome.

### 2.3 Genetic Instruments and Instrument Strength

One SNP (rs3755967, in the GC/vitamin D‐binding protein region) met conventional genome‐wide significance for circulating 25‐hydroxyvitamin D [25(OH)D] (*p* = 7.4 × 10^−9^) and is presented as a conventional‐threshold, single‐SNP (Wald‐ratio) instrument. No additional SNPs were available at *p* < 5 × 10^−8^ in the ukb‐e‐30890_CSA dataset (*n* = 7380). To allow pleiotropy‐robust sensitivity analyses (MR‐Egger regression and mode‐based estimators, which require at least three variants), two further SNPs (rs2459139, near CYP2R1; rs72744878, near DHCR7) meeting a relaxed, exploratory instrument‐selection threshold (*p* < 1 × 10^−6^) were added, yielding a three‐SNP exploratory analysis presented alongside the single‐SNP conventional analysis. Because all three SNPs were selected from the same small exposure GWAS used to estimate their effects, instrument selection at a relaxed threshold can inflate SNP–exposure effect estimates, *F*‐statistics, and *R* ^2^ for the sub‐genome‐wide‐significant SNPs (winner's curse); this is addressed as a limitation in Section 5. Instrument strength was quantified using the *F*‐statistic (*F* = [beta/SE]^2^), with *F* > 10 as the conventional threshold supporting the relevance assumption [6], *F* > 10 indicates acceptable conventional instrument strength but does not by itself establish the independence or exclusion‐restriction assumptions. The proportion of variance in Vitamin D levels explained by each instrument (*R* ^2^) was estimated as *R* ^2^ = 2 × MAF × (1 − MAF) × beta^2^, where MAF denotes the minor allele frequency and beta the SNP effect on inverse‐rank normal‐transformed (IRNT) serum 25(OH)D, the transformation applied to continuous biomarkers within each ancestry group in Pan‐UK Biobank. Total *R* ^2^ across all three instruments was 0.0106 (1.06%), substantially lower than estimates from larger European Vitamin D GWAS (typically > 5%–10% using hundreds of SNPs), which limits instrument precision and widens causal confidence intervals.

Independent SNPs were retained after linkage‐disequilibrium (LD) clumping using the Two Sample MR default parameters: *r* ^2^ < 0.001, clumping window 10,000 kb, with the 1000 Genomes Phase 3 South Asian (SAS) population as the LD reference panel. No proxy SNPs were required; all three index SNPs were directly available in the outcome GWAS. Palindromic SNPs were handled by allele frequency alignment (forward‐strand inference); no palindromic SNPs were present among the final three instruments. The genome builds for both the exposure and outcome GWAS are GRCh37/hg19, consistent with the OpenGWAS harmonization pipeline. Steiger directionality filtering was not applied, as all instruments were selected on the basis of established biological prior knowledge and their associations with circulating 25(OH)D in a prior GWAS rather than data‐driven selection alone. Table 2 shows the genetic instruments and instrument strength.

**TABLE 2:** Genetic instruments for serum 25‐hydroxyvitamin D [25(OH)D] levels (ukb‐e‐30890_CSA).

SNP	Gene/Region	Function	Beta (exposure)	SE	*p*	*R* ^2^ (%)	*F*‐statistic
rs3755967	GC	Vitamin D‐binding protein	0.099	0.017	7.4 × 10^−9^	0.405	33.4
rs2459139	CYP2R1	25‐hydroxylase	−0.108	0.020	7.3 × 10^−8^	0.342	29.0
rs72744878	DHCR7	VitD synthesis	−0.101	0.020	2.7 × 10^−7^	0.309	26.4

*Note:* rs3755967 met the conventional genome‐wide significance threshold (*p* < 5 × 10^−8^); rs2459139 and rs72744878 met a relaxed, exploratory instrument‐selection threshold (*p* < 1 × 10^−6^) used to enable pleiotropy‐robust sensitivity analyses in this South Asian GWAS (*n* = 7380). Beta values represent the change in SD units of IRNT 25(OH)D per effect allele. MAF = Minor Allele Frequency; MAF values correspond to the effect allele frequency (EAF) in the Pan‐UK Biobank South Asian GWAS. All *F*‐statistics exceed the weak instrument threshold of 10 (mean *F* = 29.6); *F*‐statistics and *R* ^2^ for the two exploratory‐threshold SNPs may be inflated by winner's curse (Sections 2.3 and 5). Total *R* ^2^ = 0.0106. Chromosomal positions are GRCh37/hg19: rs3755967, Chr4:72,609,398 (GC/VDBP locus); rs2459139, Chr3:145,449,231 (CYP2R1 locus); rs72744878, Chr1:202,033,857 (DHCR7/NADSYN1 locus).

### 2.4 MR Assumptions

Three core instrumental variable assumptions were evaluated: (1) Relevance, SNPs are robustly associated with Vitamin D levels (confirmed; all *F*‐statistics > 10); (2) Exclusion restriction, SNPs influence T2D solely through Vitamin D and not via alternative biological pathways (horizontal pleiotropy); and (3) Independence, SNPs are not associated with confounders of the Vitamin D–T2D relationship. The limited number of instruments (*n* = 3) precludes more comprehensive pleiotropy analyses such as MR‐PRESSO; the MR‐Egger intercept test has limited statistical power in this context.

### 2.5 Data Harmonization and Statistical Analysis

Data harmonization was performed to align the effect alleles across the exposure and outcome datasets. Palindromic SNPs were handled using allele frequency information to infer strand orientation. The primary causal estimate was derived using the Inverse Variance Weighted (IVW) method, with all causal effects reported per one standard deviation (SD) increase in inverse‐rank normal‐transformed (IRNT) serum 25(OH)D. Pre‐specified sensitivity analyses used MR‐Egger regression [11, 12], the Weighted Median estimator [13], and Simple and Weighted Mode estimators [14]. Heterogeneity across SNP‐specific causal estimates was assessed using Cochran's *Q* test (*Q p* < 0.05 indicating significant heterogeneity). Causal estimates are reported as beta coefficients and converted to odds ratios (OR) with 95% confidence intervals (CI). All analyses were conducted in R (version 4.3.1) [15] using the Two Sample MR package (version 0.7.0) [16].

### 2.6 Power Calculation

A post hoc power calculation was conducted using the mRnd framework [17] to quantify the study's ability to detect a clinically meaningful causal effect per 1 SD increase in IRNT serum 25(OH)D. Power was estimated at alpha = 0.05 using the outcome sample (*n* = 50,533; case fraction = 0.330) and a total *R* ^2^ = 0.0106 across a range of clinically relevant odds ratios (OR = 1.1, 1.2, 1.5).

## 3 Results

### 3.1 Instrument Strength and Variance Explained

All three genetic instruments demonstrated acceptable conventional instrument strength. Individual *F*‐statistics ranged from 26.4 (rs72744878, DHCR7 locus) to 33.4 (rs3755967, GC/vitamin D‐binding protein locus), with a mean of 29.6, above the conventional weak instrument threshold of 10. *F* > 10 supports the relevance assumption but does not establish independence or exclusion‐restriction. The total *R* ^2^ of 1.06% reflects the limited size of the available South Asian Vitamin D GWAS (*n* = 7380) and the relaxed, exploratory instrument‐selection threshold used for two of the three SNPs, which may inflate their *F*‐statistics and *R* ^2^ (winner's curse; Sections 2.3 and 5), resulting in wider confidence intervals than would be expected from a larger, more powered instrument set.

### 3.2 Heterogeneity and Pleiotropy Assessment

Cochran's *Q* test revealed no significant heterogeneity across SNP‐specific causal estimates (IVW: *Q* = 1.816, df = 2, *p* = 0.403; MR‐Egger: *Q* = 0.547, df = 1, *p* = 0.459), suggesting consistency of causal direction across instruments. The MR‐Egger intercept provided no evidence of directional pleiotropy (intercept = −0.322, SE = 0.286, *p* = 0.462). With only three SNPs, MR‐Egger has a single residual degree of freedom, so the intercept and slope estimates are extremely unstable; a non‐significant intercept should not be interpreted as reassuring evidence that horizontal pleiotropy is absent. Both tests have very limited statistical power with only three instruments, and horizontal pleiotropy cannot be fully excluded.

### 3.3 Primary MR Analysis

The primary IVW analysis found no statistically significant association between genetically predicted Vitamin D levels and T2D risk per 1 SD increase in IRNT serum 25(OH)D (*b* = 0.058, SE = 0.120, *p* = 0.630; OR = 1.06, 95% CI: 0.83–1.36) in South Asians using the BMI‐unadjusted outcome (GCST90093109). The confidence interval, while narrower than the prior analysis, still spans both protective and harmful effect directions and does not exclude moderate causal effects. This null result indicates an absence of a detectable association within the precision afforded by current instruments, rather than definitive evidence of no causal effect.

### 3.4 Sensitivity Analyses

Alternative MR estimators were statistically non‐significant, although the MR‐Egger estimate was extremely imprecise because only three instruments were available (Table 3). The Weighted Median, Simple Mode, and Weighted Mode estimates were close to the null and consistent in direction with the primary IVW estimate. The MR‐Egger estimate (OR = 32.0, 95% CI: 0.11–9103), however, was not close to the null and did not point in the same direction as the other methods; its extremely wide confidence interval reflects estimation instability rather than support for a null effect, and should not be described as part of a “consistent direction of null results.” The small number of instruments (*n* = 3) limits the reliability of MR‐Egger and mode‐based estimates individually. To further evaluate heterogeneity and pleiotropy given the limited power of tests based on only three instruments, we additionally repeated the analysis using relaxed instrument‐selection thresholds of *p* < 5 × 10^−5^ (68 SNPs) and *p* < 1 × 10^−5^ (18 SNPs). Results were directionally consistent with the primary 3‐SNP analysis across all thresholds (Table 4). With substantially more instruments, both Cochran's *Q* and the MR‐Egger intercept provided more informative and reassuring diagnostics: neither showed significant evidence of heterogeneity or directional pleiotropy (Egger intercept *p* = 0.194 and *p* = 0.685 at the 5 × 10^−5^ and 1 × 10^−5^ thresholds, respectively).

**TABLE 3:** MR results across all methods (primary outcome: GCST90093109, BMI‐unadjusted).

MR method	nSNP	Beta	SE	*p*	OR (95% CI)	Note
IVW (primary)	3	0.058	0.120	0.630	1.06 (0.83–1.36)	Primary analysis
MR‐Egger	3	3.466	2.883	0.457	32.0 (0.11–9103)	Pleiotropy‐robust
Weighted Median	3	0.060	0.139	0.669	1.06 (0.81–1.39)	≥ 50% valid SNPs
Simple Mode	3	0.252	0.159	0.253	1.29 (0.94–1.76)	Sensitivity
Weighted Mode	3	−0.076	0.136	0.631	0.93 (0.71–1.21)	Sensitivity

*Note:* Beta values represent the change in log‐odds of T2D per 1‐SD IRNT serum 25(OH)D. The MR‐Egger estimate is markedly imprecise (1 residual degree of freedom with only 3 SNPs) and is not directionally consistent with the other estimators; it should not be summarized as a null result. Cochran's *Q* heterogeneity: IVW *Q* = 1.816, df = 2, *p* = 0.403; MR‐Egger *Q* = 0.547, df = 1, *p* = 0.459. MR‐Egger intercept (pleiotropy test): Intercept = −0.322, SE = 0.286, *p* = 0.462.

Abbreviations: CI, confidence interval; IVW, Inverse Variance Weighted; OR, odds ratio.

**TABLE 4:** Sensitivity analysis using relaxed instrument‐selection thresholds.

Threshold	N SNPS	*β* (IVW)	SE	*p*	Q (IVW)	QP value	Egger intercept *p*
Original (1 × 10^−6^)	3	0.058	0.120	0.630	1.816	0.403	—
5 × 10^−5^	68	0.023	0.030	0.450	85.98	0.059	0.194
1 × 10^−5^	18	0.031	0.046	0.494	9.31	0.930	0.685

Abbreviations: IVW, Inverse Variance Weighted; Q, Cochran's *Q* heterogeneity statistic; SNP, single nucleotide polymorphism.

### 3.5 BMI‐Adjusted Sensitivity Analysis

As a pre‐specified sensitivity analysis, the same three instruments were used to estimate the causal effect of genetically predicted Vitamin D on the BMI‐adjusted T2D outcome (ebi‐a‐GCST90093110). The IVW estimate was similar in direction and magnitude to the primary analysis (*b* = 0.062, SE = 0.125, *p* = 0.619; OR = 1.06, 95% CI: 0.83–1.36). The Weighted Median (*b* = 0.060, SE = 0.144, *p* = 0.678; OR = 1.06, 95% CI: 0.80–1.41), Simple Mode (*b* = 0.252, SE = 0.157, *p* = 0.250; OR = 1.29, 95% CI: 0.95–1.75), and Weighted Mode (*b* = −0.076, SE = 0.150, *p* = 0.661; OR = 0.93, 95% CI: 0.69–1.24) were all non‐significant. The MR‐Egger estimate remained extremely imprecise (*b* = 3.466, SE = 2.883, *p* = 0.442; OR = 32.0, 95% CI: 0.11–9103). There was no significant heterogeneity (IVW: *Q* = 1.424, df = 2, *p* = 0.491; MR‐Egger: *Q* = 0.029, df = 1, *p* = 0.866) and no evidence of directional pleiotropy (MR‐Egger intercept = −0.350, SE = 0.296, *p* = 0.447). These estimates were directionally and quantitatively consistent with the primary BMI‐unadjusted analysis reported in Sections 3.3 and 3.4, providing reassurance that conditioning on BMI in the outcome GWAS did not materially alter the causal estimate.

### 3.6 Power Calculation

Given the outcome sample size (*n* = 50,533; case fraction = 33.0%) and *R* ^2^ = 0.0106, the post hoc power calculation (mRnd framework) showed approximately 18% power to detect OR = 1.1, approximately 51% power for OR = 1.2, and greater than 99% power for OR = 1.5 at alpha = 0.05. These low‐to‐moderate power estimates for smaller effect sizes are consistent with the wide observed IVW confidence interval (OR: 0.83–1.36) and reflect the limited instrument strength available from current South Asian GWAS resources. The study cannot reliably detect small‐to‐moderate causal effects, which explains why the confidence interval spans both protective and harmful directions despite the large outcome sample. Table 5 shows post hoc power calculations.

**TABLE 5:** Post hoc power calculations (mRnd framework). Outcome *n* = 50,533; case fraction = 33.0%; alpha = 0.05; *R* ^2^ = 0.0106 (entered as decimal proportion, not percentage); per 1‐SD increase in IRNT serum 25(OH)D. Low power for OR = 1.1 and moderate power for OR = 1.2 explain the wide IVW confidence interval (0.83–1.36) and reflect the limited instrument strength from current South Asian GWAS resources.

Target OR	Estimated power	Interpretation
OR = 1.1	~18%	Underpowered; cannot reliably detect small effects
OR = 1.2	~51%	Moderate power; small effects may be missed
OR = 1.5	> 99%	Well powered for large effects

### 3.7 Figures

Figure 3 shows an MR scatter plot of SNP‐specific effects on Vitamin D (*x*‐axis) versus T2D (*y*‐axis), with regression lines from each MR method overlaid. Each data point represents one genetic instrument. The near‐horizontal slope of the IVW line is consistent with the absence of a meaningful causal association. The divergence between the MR‐Egger and IVW lines reflects the small number of instruments and should not be overinterpreted in the absence of a reliable MR‐Egger intercept estimate (only one residual degree of freedom).

**FIGURE 3:**
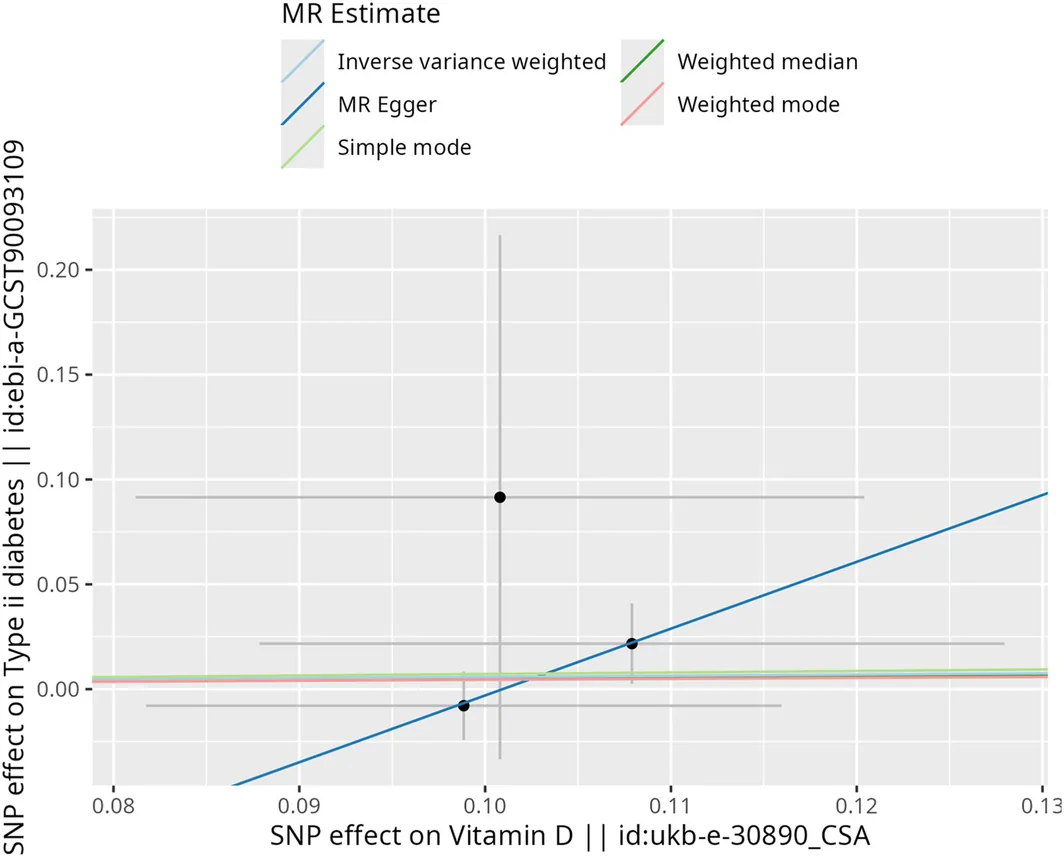
MR scatter plot. Each point represents one genetic instrument (SNP): (1) rs3755967‐GC, (2) rs2459139‐CYP2R1, (3) rs72744878‐DHCR7. *X*‐axis: SNP effect on serum 25(OH)D (ukb‐e‐30890_CSA, IRNT scale). *Y*‐axis: SNP effect on T2D (GCST90093109, BMI‐unadjusted). Error bars represent 95% confidence intervals. The near‐horizontal IVW slope is consistent with a null causal estimate; the steep MR‐Egger line reflects instability with only 3 SNPs rather than a reliable alternative estimate.

Figure 4 shows the MR funnel plot presenting individual SNP causal estimates (*x*‐axis: beta‐IV) against instrument precision (*y*‐axis: 1/SE). With only three SNPs, funnel plot asymmetry cannot be reliably assessed, and the plot should be interpreted descriptively rather than as a formal pleiotropy test.

**FIGURE 4:**
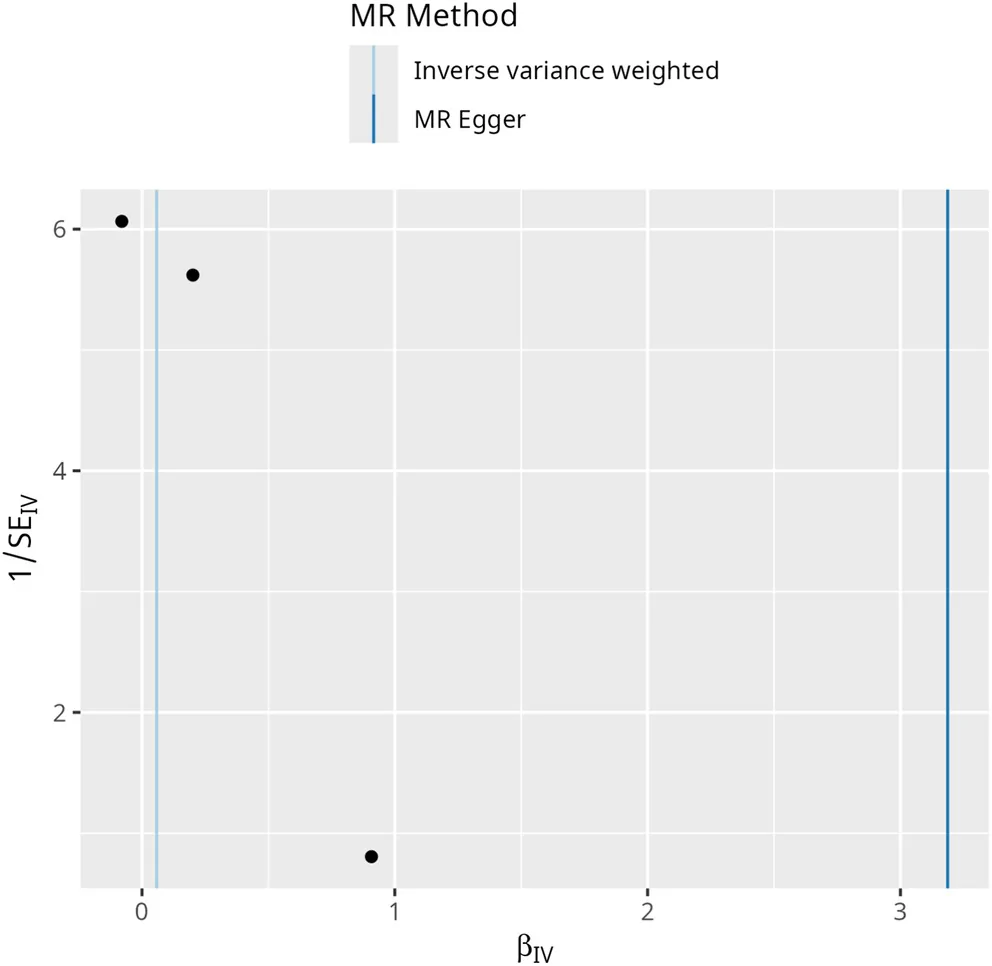
MR funnel plot. Individual SNP causal effect estimates plotted against instrument precision (*y*‐axis: 1/SE). IVW and MR‐Egger reference lines shown; the MR‐Egger line is shown for completeness but is not a reliable summary given only 3 SNPs.

## 4 Discussion

In this two‐sample Mendelian randomization study, genetically predicted Vitamin D levels were not significantly associated with the risk of Type 2 diabetes in South Asian populations. The primary IVW analysis showed no evidence of an association per 1‐SD increase in IRNT serum 25(OH)D. The Weighted Median, Simple Mode, and Weighted Mode estimates were close to the null and directionally consistent with the primary analysis; the MR‐Egger estimate, however, was extremely imprecise and pointed in a different direction, reflecting instability with only three instruments rather than corroborating evidence of a null effect. The instruments met acceptable conventional strength criteria, with all *F*‐statistics exceeding 10 (mean *F* = 29.6), although *F*‐statistics and *R* ^2^ for the two exploratory‐threshold instruments may be inflated by winner's curse. There was no significant heterogeneity across SNP‐specific estimates and no evidence of directional pleiotropy based on the MR‐Egger intercept, although both tests have very limited power with only three instruments. Taken together, these findings suggest that the inverse association between Vitamin D deficiency and Type 2 diabetes reported in observational studies may not reflect a direct causal relationship, at least within the precision limits of currently available South Asian genetic data, although small‐to‐moderate causal effects cannot be excluded [6, 7, 11, 12, 13, 14].

This result is clinically important given that both Vitamin D deficiency and Type 2 diabetes are highly prevalent in South Asian populations. Pakistan in particular carries a high burden of both conditions, and their co‐occurrence has long suggested that Vitamin D deficiency might contribute to diabetes risk [1, 3]. These findings emphasize the importance of causal inference approaches, such as Mendelian randomization, when interpreting observational associations between Vitamin D status and Type 2 diabetes risk [6, 7].

### 4.1 Comparison With Prior Studies

The findings of the present study are broadly consistent with previous Mendelian randomization evidence. Ye et al. found no significant causal effect of circulating 25‐hydroxyvitamin D on incident Type 2 diabetes in a large European‐ancestry MR analysis [8]. Similarly, Bejar et al. found no significant association between a composite Vitamin D genetic score and Type 2 diabetes risk in South Asian and European populations, though a signal from the DHCR7 locus was noted, consistent with the overall evidence remaining mostly null or inconclusive rather than definitively causal [18]. The present study adds to this literature by focusing specifically on South Asian ancestry, using South Asian Type 2 diabetes GWAS data to estimate outcomes [9] and instruments derived from a South Asian‐specific Vitamin D GWAS. The ancestry‐specific approach is important because causal estimates derived from European populations may not fully apply to South Asian populations, which differ in adiposity patterns, insulin resistance profiles, dietary exposures, skin pigmentation, and genetic architecture.

The relationship between MR and randomized‐trial evidence is more nuanced than a single null trial result suggests. The D2d trial did not specifically enrol participants who were free of Vitamin D deficiency; rather, it enrolled adults with prediabetes regardless of baseline Vitamin D status, and the investigators concluded that participants were not selected for Vitamin D insufficiency. In the primary analysis, Vitamin D3 supplementation did not significantly reduce the risk of Type 2 diabetes in this population [19]. However, a subsequent individual‐participant‐data meta‐analysis pooling D2d with two other prediabetes prevention trials (DPVD and the Tromsø/Norwegian trial) reported an adjusted 15% relative reduction in diabetes risk with Vitamin D supplementation among adults with prediabetes [20]. MR and supplementation‐trial estimates address different biological questions: MR evaluates lifelong, genetically predicted differences in Vitamin D status across the population distribution, whereas supplementation trials evaluate a clinical intervention delivered over a limited period, often in participants selected for elevated metabolic risk. The recent IPD meta‐analysis suggesting a possible modest benefit specifically in people with prediabetes does not necessarily conflict with a null population‐level linear MR estimate; the two literatures may instead point to a clinically relevant effect that is concentrated in a high‐risk subgroup rather than detectable as a linear, population‐wide causal effect.

### 4.2 Biological Interpretation and Non‐Linear Effects

There are several possible explanations for the absence of a detectable causal effect. First, Vitamin D may be a marker of poor metabolic health rather than a cause of Type 2 diabetes; people with obesity, sedentary habits, and poor diet tend to have both low Vitamin D levels and elevated diabetes risk. South Asians develop T2D at lower BMI levels than White European populations [21], suggesting that BMI may incompletely capture adiposity‐related metabolic risk in this population; incorporating South Asian‐specific BMI categories and measures of central adiposity could improve the assessment of this risk and the interpretation of BMI‐adjusted analyses. Second, the effects of Vitamin D on intermediate metabolic traits may be relatively small, and the available genetic instruments in South Asia are not powerful enough to detect them. Future studies examining continuous metabolic traits, such as fasting glucose, postprandial glucose, and HOMA‐IR, may help determine whether associations are detectable at earlier stages of dysglycaemia. Third, the causal effect may not be linear: it might only emerge at very low 25(OH)D concentrations that are not distributed across the full population range and would therefore be missed by standard linear MR methods. This is particularly relevant in South Asian populations, where severe deficiency is common. Future studies using non‐linear MR approaches and individual‐level data could help determine whether threshold‐dependent causal effects exist at the deficiency range most prevalent in South Asia. Population‐specific differences in Vitamin D receptor polymorphisms, dietary calcium cofactors, and metabolic context may also modulate any underlying causal relationship.

### 4.3 Public Health Implications

From a public health perspective, these findings are directly relevant to Vitamin D supplementation strategies targeting Type 2 diabetes prevention in Pakistan and the broader South Asian region. Supplementation programs specifically aimed at reducing the incidence of Type 2 diabetes are not supported by current causal evidence. Healthcare resources may be more efficiently directed toward interventions with established causal benefit, including lifestyle modification, dietary reform, and pharmacological prevention in high‐risk individuals. These findings do not preclude the benefits of Vitamin D supplementation for other clinically important outcomes such as bone health or immune function. Notably, the large VITAL randomized trial did not find a reduction in cardiovascular events with Vitamin D supplementation [22], so cardiovascular risk reduction should not be assumed as an established benefit. Overall, these findings suggest that correcting Vitamin D deficiency is unlikely to substantially reduce the incidence of Type 2 diabetes unless future evidence identifies a specific high‐risk subgroup that benefits [3, 19, 20].

### 4.4 Strengths

A key strength of this study is its use of a Mendelian randomization design to address a clinically relevant question in an underrepresented ancestry group. South Asians carry a disproportionate burden of Type 2 diabetes yet remain underrepresented in genetic epidemiology. The use of ancestry‐specific GWAS data reduces concerns about population mismatch and improves the relevance of the findings for South Asian populations. The analysis also used multiple MR methods with different pleiotropy assumptions, all of which produced statistically non‐significant results (though not all directionally consistent; see Section 3.4). Instrument strength was of acceptable conventional magnitude, heterogeneity was not significant, and the MR‐Egger intercept did not indicate directional pleiotropy, with the caveat that both tests have limited power with only three instruments. Reporting followed STROBE‐MR principles, improving transparency and reproducibility [10].

## 5 Limitations

Several limitations must be acknowledged. First and most importantly, the South Asian Vitamin D exposure GWAS (ukb‐e‐30890_CSA; *n* = 7380) yielded only one SNP at conventional genome‐wide significance (*p* < 5 × 10^−8^), necessitating a relaxed instrument selection threshold (*p* < 1 × 10^−6^) for two of the three instruments to enable pleiotropy sensitivity analyses, and resulting in a total *R* ^2^ of only 1.06%. This limits instrument precision and the reliability of pleiotropy tests. Although relaxed instrument‐selection thresholds enabled additional heterogeneity and pleiotropy assessments, these exploratory analyses may still be affected by winner's curse because the additional variants were selected from the same relatively small exposure GWAS. The null result should therefore be interpreted as the absence of evidence of a detectable causal effect at current instrument power, rather than evidence of no effect whatsoever.

Second, only three SNPs were available as genetic instruments, restricting the statistical power of pleiotropy tests. MR‐PRESSO and robust leave‐one‐out analyses could not be performed with adequate precision, and the MR‐Egger intercept test has very limited power given the small number of instruments. The funnel plot shows one SNP (rs72744878) with a notably divergent estimate, which cannot be formally tested for outlier status with so few instruments.

Third, the BMI‐unadjusted outcome GWAS (GCST90093109) was used as the primary analysis, with the BMI‐adjusted model (GCST90093110) as a pre‐specified sensitivity analysis, addressing the collider bias concern raised with the original analysis design.

Fourth, UK Biobank South Asian participants are UK‐resident South Asian participants who may differ systematically from populations in Pakistan due to healthy migrant selection and dietary acculturation, limiting direct generalisability; we are not aware of confirmed country‐of‐birth data for this subgroup and have avoided assuming it.

Fifth, standard linear MR cannot detect non‐linear or threshold causal effects of Vitamin D, which may be especially relevant given the high rates of severe deficiency in South Asian populations.

Sixth, the two‐sample design assumes independent exposure and outcome samples, but this could not be fully confirmed: the exposure GWAS is entirely UK Biobank‐based, and UK Biobank was one of 16 cohorts contributing to the South Asian T2D outcome meta‐analysis [9]. The exact degree of overlap could not be determined from the published report. Sample overlap in two‐sample MR can bias estimates toward the observational (confounded) association, particularly when instruments are selected using a relaxed threshold, and this possibility cannot be excluded here.

To further evaluate the potential impact of sample overlap, MRlap was considered as an approach for overlap adjustment. However, MRlap could not be successfully implemented because the LDSC covariance estimation step produced undefined (NaN) values, likely reflecting unstable heritability or covariance estimates due to the modest size of the South Asian Vitamin D GWAS (*n* = 7380). Therefore, we performed an alternative analytical assessment using the bounding approach of Burgess et al. [11], which estimates the maximum plausible bias from sample overlap without requiring LDSC‐based covariance estimation. Under a conservative worst‐case overlap proportion (*φ* = 0.146), assumed phenotypic correlation (*ρ* = −0.12), and mean instrument strength (*F* = 29.6), the estimated relative bias was approximately 0.32% (absolute bias ≈0.003 log‐OR units; approximately 5.6% of the observed IVW estimate). These results suggest that sample overlap is unlikely to materially influence the interpretation of the primary findings; however, this represents a theoretical bias assessment rather than a direct empirical correction, and residual uncertainty remains.

Seventh, because two of the three instruments were selected from the same small exposure GWAS used to estimate their effects, using a relaxed, exploratory threshold (*p* < 1 × 10^−6^), their SNP‐exposure effect sizes, *F*‐statistics, and *R* ^2^ may be inflated by winner's curse, which would tend to make the instruments appear stronger and the causal estimate appear more precise than they truly are. *F*‐statistics above 10 support the relevance assumption but do not, by themselves, establish that the independence or exclusion‐restriction assumptions hold. Full instrument‐selection and clumping details (LD *r* ^2^ threshold and window, ancestry‐specific reference panel, proxy‐SNP use, palindromic‐variant handling, genome build, and Steiger directionality testing) should be confirmed and reported in the final Methods to allow readers to fully evaluate these assumptions.

## 6 Conclusions

This exploratory two‐sample Mendelian randomization study found no statistically significant association between genetically predicted serum 25(OH)D and Type 2 diabetes risk in South Asian populations. The findings were broadly similar across the primary and sensitivity analyses. However, the limited number of genetic instruments, modest statistical power, and possible sample overlap mean that small or non‐linear effects cannot be excluded. These findings suggest that the observed relationship between low Vitamin D and Type 2 diabetes may partly reflect confounding or reverse causation. Larger South Asian GWAS are needed to clarify potential small, non‐linear, or subgroup‐specific associations.

## Author Contributions

**Adina Zaka:** conceptualization, writing – original draft, formal analysis, data curation. **Suleman Khan:** conceptualization, methodology, writing – review and editing. **Asim Shah:** writing – original draft, writing – review and editing. **Aizaz Anwar Khalid:** writing – original draft, validation, methodology. **Ibrahim Khalil:** writing – original draft, writing – review and editing, project administration, supervision. **A. B. M. Kamrul‐Hasan:** writing – original draft, writing – review and editing, supervision, data curation, visualization.

## Ethics Statement

This study is a secondary analysis of publicly available, de‐identified GWAS summary statistics. No individual‐level patient data were accessed at any stage. All source GWAS datasets were generated in accordance with appropriate ethical protocols, with informed consent obtained from participants at the time of data collection. Formal IRB approval was not required, and the study was conducted in accordance with the principles of the Declaration of Helsinki.

## Conflicts of Interest

The authors declare no conflicts of interest.

## Data Availability

All GWAS summary statistics are publicly available through the OpenGWAS database (https://gwas.mrcieu.ac.uk/). Exposure: ukb‐e‐30890_CSA. Primary outcome: ebi‐a‐GCST90093109; sensitivity outcome: ebi‐a‐GCST90093110 (PMID: 35393509; Loh M et al., Commun Biol 2022). The R analysis script and harmonized SNP‐level dataset are being deposited in a public repository (e.g., Zenodo or GitHub) prior to submission; a permanent repository link/DOI will be added here. In the interim, code and data are available from the corresponding author upon reasonable request.
